# A rare case of chronic Gianotti–Crosti syndrome: A case report

**DOI:** 10.1177/2050313X231164250

**Published:** 2023-04-14

**Authors:** Laura D Chin, Carmen Liy-Wong

**Affiliations:** 1 Division of Dermatology, Department of Medicine, University of Ottawa, Ottawa, ON, Canada; 2 Departments of Dermatology and Rheumatology, Children’s Hospital of Eastern Ontario, University of Ottawa, Ottawa, ON, Canada

**Keywords:** Gianotti–Crosti syndrome, acrodermatitis, papular acrodermatitis of childhood, acrodermatitis, acrodermatitis papulosa infantum, infantile papular acrodermatitis

## Abstract

Gianotti–Crosti syndrome, also known as papular acrodermatitis of childhood, is a common, self-limiting dermatosis often seen in children with triggers including viral and bacterial infections along with immunizations. Lesions are generally described as asymptomatic, skin colored to erythematous papules and papulovesicles that often spontaneously resolve within weeks. Here, we will discuss Gianotti–Crosti syndrome and present a rare case of chronic Gianotti–Crosti syndrome in an otherwise healthy 3-year-old male persisting for over 20 months. From this report, we aim to better educate the dermatologic community on the extremes of the Gianotti–Crosti syndrome disease course to improve diagnosis and treatment of symptomatic patients.

## Introduction

Gianotti–Crosti syndrome (GCS) is a relatively common viral dermatosis often seen in children, most commonly affecting individuals between 1 and 6 years of age.^1,2^ There are also numerous reports of GCS in adults, though this is a much rarer occurrence.^2,3^ GCS occurs more commonly during spring and summer seasons, as well as in atopic individuals.^2,4^

Common viral triggers include Epstein–Barr virus (EBV), cytomegalovirus (CMV), coxsackievirus, hepatitis A, B, and C viruses, along with several others. Although hepatitis B virus was originally described as the most common trigger for GCS, EBV has surpassed hepatitis B virus and has now been established as the most common cause of GCS.^2^ Bacterial infectious have also been identified as more rare GCS triggers, including *Bartonella henselae, Mycoplasma pneumoniae*, and *β-*hemolytic streptococci.^2^ In addition, a link between immunizations and GCS has been found. Some identified vaccine triggers include the Japanese encephalitis vaccine, hepatitis A and B vaccines, diphtheria-tetanus vaccine, diphtheria-tetanus-pertussis vaccine, measles-mumps-rubella vaccine, Haemophilus influenza B vaccine, and varicella zoster vaccine.^2,5^

The exact pathogenesis of GCS is not entirely known, though two hypotheses are commonly discussed. The first is that GCS is a delayed type 4 hypersensitivity reaction within the dermis secondary to various viral and bacterial pathogens as well as vaccines.^1,2,6^ The second proposed hypothesis is that an infectious trigger in combination with a secondary immunomodulatory event, such as increased IgE seen in atopic individuals, is necessary for GCS development.^2,6^

GCS presents clinically with the rapid onset of monomorphic skin colored to erythematous papules and papulovesicles. The lesions are commonly distributed symmetrically on the face, extensor surfaces of extremities, and buttocks. The lesions are often asymptomatic but can be pruritic. Truncal lesions can be present and should not exclude a diagnosis.^7^ Systemic findings in GCS may include malaise, fever, diarrhea, lymphadenopathy, and upper respiratory symptoms. Hepatosplenomegaly is rare.

Histopathologic findings are often non-specific, with findings including epidermal spongiosis with vesicles containing predominantly Langerhans cells, mild acanthosis and edema of the papillary dermis, and mixed inflammatory perivascular infiltrate, hyperkeratosis and parakeratosis of the upper dermis. CD4^+^ and CD8^+^ T-cells are the predominant lymphocytes seen with immunohistochemical analysis.^1,2^

GCS is often a self-limiting disease, resolving spontaneously within 10–60 days,^1,2^ though rapid cases of GCS resolving within 5 days and chronic cases lasting up to 12 months have been reported.^7,8^ Treatment of GCS is not always necessary given its self-limiting time course; however, topical corticosteroids and oral antihistamines might provide some symptomatic relief of pruritus. Treatment with topical steroids or oral antihistamines does not shorten the course of illness.^9^

## Case report

Here, we present a case of chronic GCS persistent for over 20 months in an otherwise healthy 3-year-old male.

After being diagnosed with hand-foot-mouth disease in August 2019, the patient began to develop a secondary cutaneous eruption. In December 2019, he was first seen by Dermatology and formally diagnosed with GCS. The rash temporarily improved with the use of betamethasone valerate 0.1% cream, which was later switched to tacrolimus 0.1% ointment due to concerns of prolonged topical corticosteroid use in addition to cetirizine for management of pruritis.

He re-presented to Dermatology in December 2020 with concerns of ongoing rash flares. The patient’s lesions persisted with intermittent improvement and exacerbation. The rash primarily affected the face and the dorsal aspects of the hands bilaterally. The lesions were largely asymptomatic though occasionally mild-to-moderately pruritic. The patient was otherwise asymptomatic and systemically well.

**Figure 1.**
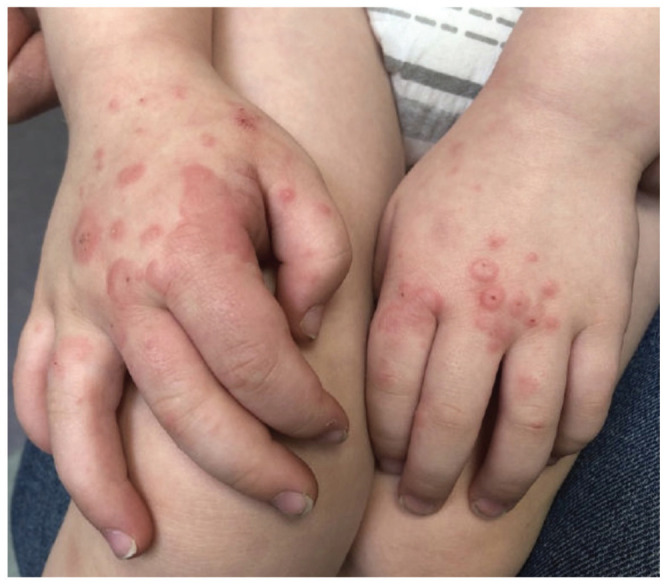
Discrete and coalescing erythematous papules with central erosion on the patient’s dorsum hands.

On physical examination, the patient had multiple erythematous papules with some erosions. Areas involved included the bilateral cheeks and dorsal aspects of hands (Figure 1). Lesions would sometimes also occur on the nose and forehead.

His blood work revealed normal complete blood count, liver enzymes and liver function tests, and reactive EBV IgG indicating past EBV infection. IgM was non-reactive. CMV, hepatitis B, and hepatitis C were non-reactive. Skin punch biopsy was consistent with GCS, showing minimal epidermal spongiosis, focal papillary dermal edema and a moderated mixed perivascular mononuclear infiltrate in addition to very rare eosinophils.

The patient’s topical treatment regime was switched to desonide 0.05% ointment for management of more severe flares and pimecrolimus 1% cream for maintenance. Most recent follow-up in July 2021 revealed well-managed disease; however, halting the use of topical treatments would result in quickly developing lesion recurrence. We suspect that recurrent lesions seen most recently reflect a post-infectious inflammatory process rather than active infection.

## Discussion

Although GCS has been reported to last as long as 12 months,^2,7^ this is a very rare occurrence. To our knowledge, there have been no reports in the literature of GCS persisting longer than 12 months. In our case of an otherwise healthy 3-year-old male, coxsackievirus infection as the likely trigger for his GCS onset in 2019. Given his reactive EBV IgG, it is possible that the patient subsequently experienced a self-limited course of EBV which propagated his GCS disease course. It is also possible that routine immunization had a role in the patient’s continued GCS disease course.

In summary, we describe a rare case of chronic GCS lasting longer than 20 months in a 3-year-old male. This atypical GCS disease course is suspected to have originated due to a coxsackievirus and subsequently prolonged due to self-limiting EBV infection or vaccination. The reviewed literature reveals no cases of GCS lasting longer than 12 months. Disease management for the patient has been successful, though lesion flares persist when topical treatment is tapered. From this report, we aim to better educate the dermatologic community on the extremes of the GCS disease course to improve diagnosis and treatment of symptomatic patients.
